# A Rare Case of Dilated Cardiomyopathy, Focal Segmental Glomerulosclerosis, and Bell’s Palsy in a 29-Year-Old Male After Coxsackievirus Infection

**DOI:** 10.7759/cureus.26285

**Published:** 2022-06-24

**Authors:** Rafsan Ahmed, Amirhossein Moaddab, Syed W Hussain, George Viriya, Suzette Graham-Hill

**Affiliations:** 1 Department of Internal Medicine, State University of New York Downstate Health Sciences University, Brooklyn, USA; 2 Department of Cardiology, State University of New York Downstate Health Sciences University, Brooklyn, USA; 3 Department of Cardiology, Kings County Hospital Center, Brooklyn, USA

**Keywords:** dilated cardiomyopathy, nephrotic syndrome, heart failure with reduced ejection fraction, focal segmental glomerulosclerosis (fsgs), bells palsy, coxsackie myocarditis, coxsackie virus

## Abstract

Dilated cardiomyopathy (DCM) is a severe myocardial disease with diversified etiologies. Coxsackievirus serotype B (CV-B) is a known cause of infectious myocarditis that leads to DCM. The pathogenesis of CV-B myocarditis is complex and involves a combination of tissue destruction from viral proliferation and host immune response. Diagnosis is based on clinical findings and the presence of post-infection elevated titers of IgM antibodies to CV-B. Echocardiography is an important imaging modality that plays a key role in diagnosing DCM. Rare complications of coxsackievirus infection may include facial paralysis and chronic kidney disease with nephrotic syndrome. Here we present a rare case of a 29-year-old-male with recent Bell’s palsy who presented with new-onset heart failure with left ventricular ejection fraction of 5% and focal segmental glomerulosclerosis nephrotic syndrome in the setting of elevated antibodies to CV-B.

## Introduction

Dilated cardiomyopathy (DCM) is a myocardial disease characterized by enlargement and dilation of one or both ventricles with impaired contractility defined as left ventricular ejection fraction (LVEF) of <40% [1]. The prevalence of DCM in the general population is 36 cases per 100,000 and accounts for approximately 10,000 deaths and 46,000 hospitalizations each year in the United States [2,3]. Patients may present with symptoms of overt heart failure, cardiac arrhythmias, thromboembolic complications, or even sudden death [4]. Etiologies of DCM are diversified and include genetic, infectious, ischemic, medication-induced, infiltrative, and most commonly idiopathic causes [5].

Myocarditis is an inflammatory condition of the myocardium, which can lead to DCM. It can result from a wide spectrum of infectious causes that include viral, such as coxsackievirus serotype B (CV-B), parvovirus B19, adenovirus, and HIV; bacterial, such as *Corynebacterium diphtheriae*, *Staphylococcus aureus*, *Borrelia burgdorferi*, and *Ehrlichia* species; protozoal, such as *Babesia*; and trypnosomal, such as *Trypanosoma cruzi* [6]. The pathogenesis of viral myocarditis is initiated by either introduction of a new virus from a pathogenic strain or reactivation of a dormant virus. The virus proliferates in the permissive tissues of the host and then travels to the myocardium via hematogenous or lymphangitic spread. Once the virus arrives at the target cells, it uses its specific receptor complex for cell entry, e.g., coxsackievirus uses coxsackie-adenoviral receptor. Damage to myocardial tissue occurs because of viral proliferation within the cardiac myocytes and because of the host’s immune response to the viral trigger [6,7]. Cellular immunity has also been shown to play a role in the development of DCM [8]. By using strategic gene targeting, Opavsky et al. were able to show that both CD4+ and CD8+ T lymphocytes can contribute to myocarditis and mortality after CV-B infection [9].

CV-B is associated with 25-40% of acute myocarditis and DCM in infants and young adults [10,11]. Few reports exist of DCM secondary to CV-B myocarditis in adults. Here we present a case of a 29-year-old-male who was admitted for new-onset heart failure with an LVEF of 5% and was found to have positive coxsackievirus A (CV-A) and CV-B antibodies.

## Case presentation

A 29-year-old Afro-Caribbean male with history of obesity, hypertension, stage 5 chronic kidney disease (CKD), and Bell's palsy, diagnosed three months ago, presented in November of 2021 to the emergency department with three days of chest tightness and progressively worsening dyspnea on exertion for approximately one month. He denied any recent illness or sick contacts. He was unaware of his CKD and denied any use of NSAIDs or herbal supplements. He reported that his father had end-stage renal disease (ESRD) in his 30s and underwent dialysis for a few years prior to renal transplant. The patient is unaware of what caused his father’s ESRD.

Initial vitals were as follows: blood pressure of 150/95 mmHg, heart rate of 103 bpm, respiratory rate of 18 breaths per minute, SpO_2_ 95%, and temperature of 98.3°F. On physical examination, the patient was in respiratory distress. He had fine bilateral inspiratory crackles, which were more prominent in the lower lung bases. His initial laboratory values on admission are shown in Table 1. His chest X-ray showed bibasilar airspace opacities, greater on the left than right. EKG demonstrated sinus tachycardia at 108 bpm, QTc of 469 ms, and T-wave inversions in V4-V6. The patient was admitted to medicine for acute decompensated heart failure.

**Table 1:** Initial laboratory values on admission.

Laboratory test	Value on admission	Reference range
Blood urea nitrogen (mg/dL)	70	6–20
Creatinine (mg/dL)	6.33	0.6–1.3
Troponin (ng/mL)	0.042	0–0.04
Brain natriuretic peptide (pg/mL)	11,883	<100
White blood cells (× 10^3^/microL)	9.91	3.8–10.4
Hemoglobin (g/dL)	11.6	13.2–16.6
Thyroid-stimulating hormone (mIU/ml)	1.43	0.5–5
Urine protein (mg/dL)	≥300	<150

**Figure 1:**
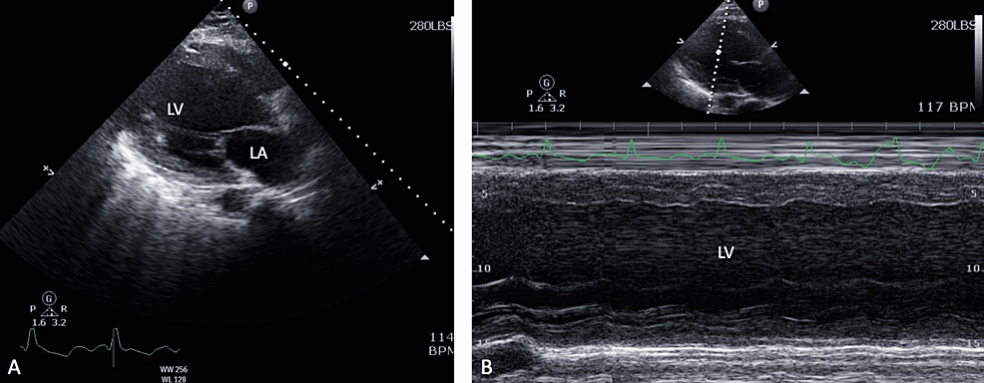
(A) Parasternal axis view of TTE showing severely dilated LA and LV. (B) M-mode showing severely dilated LV with poor LV function. TTE, transthoracic echocardiogram; LA, left ventricle; LV, left ventricle

**Figure 2:**
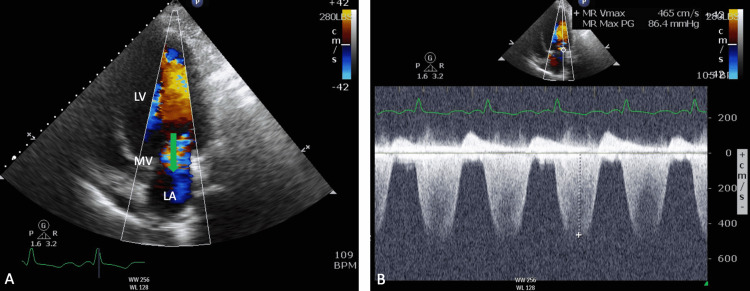
Apical two-chamber view from TTE showing severe mitral regurgitation jet (green arrow) from LV to LA. (B) Continuous wave Doppler of the regurgitant jet with low velocity and a triangular profile, suggestive of severe MR. TTE, transthoracic echocardiogram; LA, left ventricle; LV, left ventricle; MV, mitral valve; MR, mitral regurgitation

**Figure 3:**
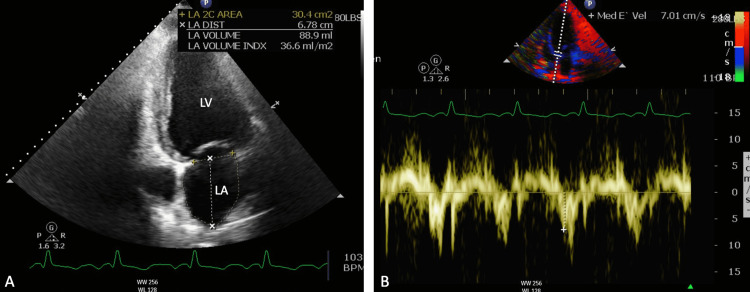
(A) Apical two-chamber view from TTE showing dimensions of LA and LV. (B) Mitral annulus motions from the apical two-chamber view with tissue Doppler imaging showing severe MR. TTE, transthoracic echocardiogram; LA, left ventricle; LV, left ventricle; MR, mitral regurgitation

Transthoracic echocardiogram (TTE) performed was significant for severely dilated left ventricle (LV) with ejection fraction (EF) of 5%, grade III LV diastolic dysfunction, severely hypokinetic wall motion, severe mitral regurgitation, and mildly dilated left atrium (Figures 1-3). Renal ultrasound showed diffuse increased echogenicity of bilateral kidneys. Cardiology and Nephrology services were consulted. He was started on furosemide, carvedilol, hydralazine, and isosorbide dinitrate for the management of his heart failure and was transferred to a different facility for cardiac catheterization.

Further lab work was remarkable for positive CV-A and CV-B antibodies. *Trypanosoma cruzi* antibody was negative. SPEP (serum protein electrophoresis) showed elevated kappa:lambda ratio of 2.05, and UPEP (urine protein electrophoresis) showed an elevated total urine protein of 334 g/L. After transfer to a different facility, the patient underwent right heart catheterization, which was notable for normal biventricular filling pressures, reduced cardiac index (CI) of 1.8, and negative pulmonary arterial hypertension. Left heart catheterization showed angiographically normal coronary arteries. Renal biopsy was significant for focal segmental glomerulosis (FSGS) in 70% of the sample. The patient was fitted with a LifeVest and tentatively planned for peritoneal dialysis in the setting of systolic heart failure.

## Discussion

Coxsackievirus belongs to the *Enterovirus* genus of the *Picornaviridae* family. They are non-enveloped viruses with positive-sensed, single-stranded RNA that are transmitted enterically [12]. CV-B is associated with a broad spectrum of clinically relevant diseases, including acute and chronic myocarditis, myopericarditis, pericarditis, DCM, meningitis, pancreatitis, hepatitis, and possibly autoimmune diabetes [13,14]. In an epidemiological study by Gaaloul et al., CV-B human infectious heart diseases were found to be more prevalent in young male adults during the autumn-winter seasons [14]. CV-A is implicated in infections of the central nervous system (e.g., aseptic meningitis), herpangina, and hand, foot, and mouth disease. CV-A has the highest incidence in males between the ages of 0-5 years during the summer-autumn seasons [15]. Approximately 10-15 million symptomatic enterovirus (EV) infections occur each year in the United States [16]. Around five million EV infections are attributed to CV-B (serotypes 1 - 5), among which 12% have myocardial involvement with serotypes 1, 3, and 5 being the most implicated [17,18].

DCM is a challenging myocardial disease; it is the most common cause of congestive heart failure (CHF) and is a major indication for heart transplantation. The estimated prevalence of DCM is approximately 1:250-400 and up to 1:2,500 in the general population [19,20]. It has no known cure or specific causes, and the five-year survival rate for patients with DCM is less than 50% [21]. Coxsackievirus serotype 3 (CV-B3) is a well-known etiology of infectious myocarditis leading to the development of DCM; around 50% of patients with DCM have antibodies reactive to CV-B3 [22,23]. The pathogenesis of CV-B3 mediated myocarditis is complex. The disease course involves an acute phase (14-18 days post-infection) and a chronic phase (beyond 18 days post-infection). During the acute phase, CV-B3 replication leads to myocardial damage through apoptosis and necrosis of cardiomyocytes. Inflammation persists in the chronic phase; however, the extent of viral replication is significantly reduced. The mechanism of myocardial injury during the chronic phase is elusive and remains unclear [24-28]. Cardiomyocyte injury is further potentiated by cytokines released from cells of the innate and adaptive immune systems. Once immune-mediated damage sets in, the disease process becomes permanent [29]. Although there are limited data on the extent of troponin elevation in cases of CV-B myocarditis, it is reasonable to attribute the minimally elevated troponin observed in our case to some element of inflammatory cardiomyopathy. It is also possible that the troponin elevation was from demand ischemia as a result of decompensated heart failure. Therapeutic strategies for CV-B3 myocarditis and DCM include antivirals, interferons, intravenous immunoglobulin (IVIG), and immunosuppressive agents such as corticosteroids and azathioprine [30-32].

Although non-specific, echocardiography provides a non-invasive and quick imaging modality that aids in the diagnosis of CV-B myocarditis by assessing ventricular size and function, EF, wall thickness, and presence of abnormal tissue Doppler profiles, and detecting pericardial effusion [33]. Several studies have shown that the principal supporting evidence for CV-B myocarditis leading to idiopathic DCM is the presence of raised titers of immunoglobulin M (IgM) antibodies against CV-B in patients with DCM compared with controls [34,35]. Here we have presented a case of a young adult male with signs and symptoms of heart failure; subsequent echocardiography showed severely dilated LV with EF of 5% in the setting of raised CV-B IgM antibodies. With no other explanations for the patient’s condition, a presumptive diagnosis of DCM from CV-B myocarditis was made. Our patient was only treated with medications for his heart failure. IVIG has anecdotal evidence in the treatment of myocarditis with no randomized trials evaluating its efficacy, and as a result, he did not undergo any immunosuppressive therapy [30].

It is also important to note that our patient was diagnosed with Bell’s palsy for which coxsackievirus is a known infectious etiology [36]. CV-A is observed to cause generalized myositis leading to flaccid paralysis, whereas CV-B causes spastic paralysis due to degeneration of neuronal tissue and focal muscle injury [37]. Mertens et al, showed the presence of neutralizing antibodies to CV-B serotype 4 in patients with facial palsy [38]. It is likely that CV-B was the culprit for our patient’s Bell’s palsy. Additionally, the presence of FSGS nephrotic syndrome is also a significant finding. CV-B causes injury to renal tissue [39]. In vitro studies show that inoculation of human podocytes and proximal tubular epithelial cells with CV-B serotypes 1-6 leads to cytopathic effects and lysis of cells [40]. Furthermore, CV-B serotype 4 injection into mice leads to mesangioproliferative glomerulonephritis and IgA nephropathy [41,42]. Zhu et al. reported a rare case of recurrent collapsing glomerulopathy, a severe subset of FSGS, associated with a relapsed coxsackievirus infection [43]. The exact pathophysiologic mechanism of kidney damage by CV-B remains unclear.

## Conclusions

Coxsackievirus is ubiquitous in nature. The burden of cardiovascular disease, specifically DCM, from CV-B infection is significant. The management of DCM is challenging; with no cure, a cardiac transplant is often indicated. Much is known about the pathophysiology of coxsackievirus myocarditis from mice and human models. However, no randomized clinical trials exist on therapeutic strategies. Some anecdotal evidence exists that supports the use of antivirals and immunosuppressive agents. Echocardiography is important in diagnosing DCM, and guideline-directed medical therapy is beneficial in the management of subsequent CHF. Additionally, coxsackievirus has serious implications for the central nervous system and the renal system. We emphasize the need for further research on the pathogenesis of this virus in different systems, randomized trials on its treatment and the development of vaccines for primary prevention.
